# The integration of urban and rural medical insurance to reduce the rural medical burden in China: a case study of a county in Baoji City

**DOI:** 10.1186/s12913-018-3611-y

**Published:** 2018-10-19

**Authors:** Pei Liu, Wei Guo, Hao Liu, Wei Hua, Linping Xiong

**Affiliations:** 1 Department of Mathematics and Physics, Second Military Medical University, Shanghai, 200433 China; 2 Department of Health Statistics, Second Military Medical University, Shanghai, 200433 China; 3 Department of Health Service, Second Military Medical University, Shanghai, 200433 China

## Abstract

**Background:**

In 2003, the Chinese government launched the New Rural Cooperative medical System(NRCMS) for its rural population. In 2007, the Urban Resident Basic Medical Insurance Scheme(URBMS) was inaugurated, which aimed to cover all urban residents who are out of the labor market. However, the accessibility and fairness of the healthcare service have hindered the progress of universal healthcare. At the beginning of 2010, the Integration of Urban and Rural Medical Insurance Scheme(IURMIS) was implemented to bridge the gap in medical care between urban and rural areas. The main objective of this study is to determine the extent to which the IURMIS has been successful.

**Methods:**

The statistical software packages SPSS 19.0 and STATA 12.0 were used for all analyses, and *P* < 0.05 was set as the required level of significance. Data were collected from outpatients from 2009 (July to December, *n* = 20,459) through 2011 (*n* = 65,258 in 2010, *n* = 59,036 in 2011) and from inpatients in 2011 (*n* = 3662). Due to the enormous amount of data and the short time span, most of our analysis was descriptive. However, regression discontinuity (RD) and the chi-squared (χ^2^) test were used to compare the ratios of medical expenses before and after the IURMIS.

**Results:**

In the RD analysis, there was a downward trend in the mean medical expense (Coef. = − 0.66, *P* < 0.001), and rural outpatients flowed to township hospitals and village clinics after the implementation of the IURMIS (Coef. = − 0.45, *P* < 0.001). However, compensation expenses showed an upward trend (Coef. = 11.59, *P* < 0.001). In the analysis of inpatient expenses, the average expenses (CNY 2067) and hospitalization days (10.0) for all the hospitals were less than those in the Chinese Fourth National Health Services Survey (CNY 3412 and 10.3, respectively).

**Conclusions:**

Rural residents’ healthcare options and quality were improved and medical expenses were significantly reduced after implementation of the scheme. These results provide an evidence-based reference for improving the integration of the urban and rural medical security systems throughout China.

## Background

Over the last few decades, China has begun to reform its healthcare system nationwide due to pressures such as an aging population and an increasing demand on health services. The Chinese government established medical insurance schemes for urban employees and retirees (namely, the Urban Employee Basic Medical Insurance Scheme (UEBMI)) in the late 1990s.

In 2003, the Chinese government proposed the New Rural Cooperative Medical System (NRCMS) to resolve the difficulties with and the high cost of medical care in rural areas, improve the health status of rural residents, and reduce rural poverty caused by poor health [1, 2]. In 2012, urban residents paid 5.38% of their per capita income out of pocket for healthcare, while their rural counterparts paid 10.76% [3]. By the end of 2014, 736 million people across China had enrolled in the NRCMS, which covered 98.9% of the target population. The number of beneficiaries who were reimbursed increased from 1745 million in 2012 to 1942 million in 2013 [4].

In July 2007, the Urban Resident Basic Medical Insurance Scheme (URBMI) was inaugurated in 79 cities and aimed to cover urban residents who are out of the labour market. By the end of 2015, 95% of the Chinese population was covered by the Chinese healthcare insurance schemes. The detailed financing and reimbursement policies and the benefit packages of the basic medical insurance schemes in China in 2014 are summarized in Table 1.

**Table 1:** The various health insurance schemes in China

Items	UEBMI	URBMI	NRCMS
Inception year	1998	2007	2003
Enrolment unit/type	Individual/andatory	Individual/Voluntary	Individual/Voluntary
Eligible population (millions)	393.1	356.1	744.2
Number of people (millions)	283.0	314.5	736.0
Population coverage (%)	72.0	88.3	98.9
Source of funding	Employer (6%) + employee (2%) contribute from the employee’s total bill in the previous year; retired workers are exempted from premium contributions.	CNY 410 per person per year for residents (including government contributions)	CNY 410 per person per year for residents (including government contributions)
Benefit packages	*INPATIENT & CRITICAL OUTPATIENT* • Deductible: Approximately 10% of local employees’ annual average wages • Reimbursement rate: 70–95% • Ceiling: 4 times the local employees’ annual average wages	*INPATIENT & CRITICAL OUTPATIENT* • Deductible: CNY 540-CNY 980 • Reimbursement rate: 50–75% • Ceiling: CNY 16,000 per person per year	*INPATIENT & OUTPATIENT* (Note: Deductible, reimbursement rate and ceiling are varied across cities/counties and medical institute levels)
Out-of-pocket health expenditures	32% (approximately CNY 3235 for urban, CNY 1274 for rural)		

Source: Ministry of Human Resources and Social Security of the People’s Republic of China, 2015-5-28

National Health and Family Planning Commission of the People’s Republic of China, 2015-11-5

National Bureau of Statistics of the People’s Republic of China, 2015-2-26

However, China’s medical security system has different patterns in urban and rural areas, including patterns due to regional segmentation. Therefore, it is important that urban and rural areas become more similar so that rural residents have equal opportunities to receive medical services and so that utilization of medical institutions can be improved and the ability of medical insurance providers to avoid risk can be enhanced [5]. Compared with urban residents, rural populations are generally younger, and younger people tend to be healthier [6]. This will help increase the medical insurance funds for current and future conditions. To improve equality between urban and rural areas, the Chinese government selected some of its provinces and cities as pilot areas for the Integration of Urban and Rural Medical Insurance Scheme (IURMIS) with the goal of establishing a unified medical insurance system. Success in the pilot areas would result in the IURMIS being extended throughout the country.

Baoji, a city that is important to the strategic development of western China, was chosen as one of the pilot cities for public hospital reform. Baoji features nearly full health coverage of its urban and rural residents, basic policy experience and practical experience in promoting the reforms of public hospitals. All these efforts have helped provide insurance coverage to its citizens.

This study examines Feng County in Baoji City in Shaanxi Province, which implemented the IURMIS at the beginning of 2010, as a case study. The county had a government-run voluntary insurance system for its residents, who received higher reimbursements if they had paid higher premiums. It merged the URBMS with the NRCMS [7–11]. After the pilot scheme began, the gap between urban and rural areas in receiving medical services and medical insurance narrowed.

This indicated that the scheme was equitable with regard to the accessibility of medical services, meaning that rural residents had opportunities to receive medical services that were equal to those of urban residents. In this case, the IURMIS covered more than 99% of the target population. Feng county, which is located in the southwest of Baoji City, mostly complied with Baoji’s medical policy. The detailed financing and reimbursement policies and the benefit packages of the basic medical insurance schemes in Baoji City in 2014 are summarized in Table 2.

**Table 2:** The various health insurance schemes in Baoji City

Items	UEBMI	URBMI	NRCMS
Inception year	1999	2007	2004
Enrolment unit/type	Individual/Mandatory	Individual/Voluntary	Individual/Voluntary
Eligible population (thousands)	536.4	483.6	2733.7
Number of people (thousands)	526.9	476.1	2728.2
Population coverage (%)	98.2	98.4	99.8
Source of funding	Employer (6%) + employee (2%) contribute from the employee’s total bill in the previous year; retired workers are exempted from premium contributions.	CNY 400 per person per year for residents (including government contributions)	CNY 230 per person per year for residents (including government contributions)
Benefit packages	*INPATIENT & CRITICAL OUTPATIENT* • Deductible: Approximately 10% of local employees’ annual average wages • Reimbursement rate: 70–95% • Ceiling: 4 times the local employees’ annual average wages	*INPATIENT & SOCIAL POOLING FOR CATASTROPHIC DISEASE* • Deductible: CNY 150-CNY 600 • Reimbursement rate: 60–90% • Ceiling CNY 30,000 per person per year	*INPATIENT & SOCIAL POOLING FOR CATASTROPHIC DISEASE* • Deductible: CNY 180-CNY 1500 • Reimbursement rate: 45–85% • Ceiling: CNY 30,000 per person per year *OUTPATIENT* • Reimbursement rate: 55–65% • Ceiling: 40*number of insured per household
Out-of-pocket health expenditures	34.1% (approximately CNY 2978 for residents)		

Source: Baoji Bureau of Statistics, 2015-3-20

Baoji People’s Government, 2014-6-26

However, the effects of the NRCMS differed in that newer households consumed fewer services than some households that had participated in the system for longer periods (more than 1 year) [12]. Many studies of the NRCMS have suggested that increasing numbers of Chinese rural residents benefit from the system [13–15], but the studies of the IURMIS and its benefits have been insufficient. Research on these aspects of China’s healthcare system has been limited to studies of policy theory [16–18], and the necessary empirical support has been lacking. In this context, this study focuses on rural residents in Feng County, their treatment patterns, and changes in the distribution of medical expenses before and after the implementation of the IURMIS to provide an evidence base from which to improve the security of the integration of the urban and rural medical systems. The study mainly focuses on the changes in medical expenses in the county before and after the implementation of the IURMIS. The outpatient variables examined include the average outpatient expenses, reimbursements, and patient flow. The inpatient variables examined include the average inpatient expenses and hospitalization days.

## Methods

### Sample

Baoji City, which is located in the middle of Shaanxi Province, is the second largest city in that province and has a population of 3.72 million. Its population consists mostly of rural residents, who account for 78.27% of the total population [19]. The per capita income of urban residents was CNY 29,475 in 2015, whereas that of the rural residents was CNY 9511 [20].

The study site was Feng County, which is located in the southwest of Baoji City in Shaanxi Province. By the end of 2015, there were 0.11 million people in this county. The rural residents accounted for 53.88% of the population. According to the Sixth National Census [21], the age distribution of the population of Feng County is similar to that of Baoji City. The population younger than 14 years were 13.14% in Feng County and 14.32% in Baoji City, and the population between 15 and 59 years old were 73.61% and 73.08%, respectively. The population aged 60 years or older accounted for 13.25% and 12.60% of the total. The per capita income of the urban residents of Feng County was CNY 30,432 in 2015, whereas that of the rural residents was CNY 10,236 [22].

The data used in this study were collected by all the primary medical institutions that provided basic public health services and basic medical services, such as treatment for common diseases, to residents of Feng County at the county, township, and village levels. The data were collected through the daily outpatient and inpatient information system and then summarized by the information department of the Health Bureau of Baoji City, Shaanxi Province, and analysed anonymously. The data mainly consisted of outpatient data from 2009 (July to December, *n* = 20,459) to 2011 (*n* = 65,258 in 2010, *n* = 59,036 in 2011). Because data from the first half of 2009 were missing, we used the data from the second half of 2009 to replace them. We used the data from the same period in 2010 to estimate the missing data for the county hospital in the third quarter of 2011. At the same time, inpatient data for 2011 were also used (*n* = 3662). After removing duplicates and incomplete records, there were a total of 3500 records.

### Demographic characteristics

This section contained information on the gender, ethnicity, and age of the outpatients and inpatients.

### Outpatient characteristics

This section included outpatient prescription medications, treatment details, and outpatient expenditures.

### Inpatient characteristics

This section included medical expenses for hospitalization, expense distributions, admission dates, discharge dates, and diagnoses of diseases. Data from the 2008 National Health Survey [23] and the 2012 Chinese Health Statistics Yearbook were also used [24].

### Measures

For both outpatients and inpatients, the patient’s age was considered the age on the date of service in years.

### Relevant definitions

According to the Chinese census register standard, we classified agriculture registered permanent residence as the **rural population**, and non-agriculture registered permanent residence as the **urban population**.

**Burden** refers to the heavy economic burden of the disease, that is, the proportion of the medical expenses attributed to the residents represents a high proportion of the total household expenditures.

**Benefit** means benefits for the insured people. In this paper, we considered decreased medical expenses and increased compensation as benefits for the insured population.

**Reimbursement rate** is defined as the proportion of the reimbursement amount to the actual amount for outpatients.

**Compensation rate** is defined as the proportion of the number of outpatients from medical reimbursement to the actual number of outpatients.

**Government contribution** is defined as the proportion that comes from the government finances.

**Deductible** means the standards of basic medical security. In an insurance policy in China, the deductible is the amount that must be paid out of pocket by the policyholder before an insurance provider will pay any expenses.

China’s administrative units are currently based on a three-tier system, dividing the nation into **provinces, counties and townships**. The country is divided into provinces, autonomous regions and **municipalities** directly under the Central Government. A province or an autonomous region is subdivided into autonomous prefectures, **counties**, autonomous counties and **cities**. A county or an autonomous county is subdivided into **townships**, ethnic townships and towns.

According to the Hospital Classification System issued by Ministry of Health of People’s Republic of China, hospitals in China are classified into primary (tier-1), secondary (tier-2) and tertiary (tier-3) institutions, which is known as the **hospital level** in China [25].

**Patient flow (%)** is defined as proportion of the number of patients at the different levels of hospitals to the number of actual patients in the corresponding year.

**Primary medical institutions** are typically a township hospital that contains less than 100 beds. They are tasked with providing preventive care, minimal health care and rehabilitation services. **Secondary hospitals** tend to be affiliated with a medium size city, county or district and contain more than 100, but less than 500 beds. They are responsible for providing comprehensive health services, as well as medical education and conducting research on a regional basis. **Tertiary hospitals** round out the list as comprehensive or general hospitals at the city, provincial or national level, with a bed capacity exceeding 500. They are responsible for providing specialist healthcare services and perform a bigger role with regard to medical education and scientific research.

### Statistical analysis

SPSS 19.0 and STATA 12.0 were used for all analyses, and *P* < 0.05 was set as the required level of statistical significance. Due to the enormous volume of data and the short time span, we performed a predominantly descriptive analysis that included frequencies, percentages, and other descriptive statistics.

Regression discontinuity (RD) and the chi-squared (*χ*^2^) test were used to compare the ratios of medical expenses before and after the implementation of the IURMIS. Because medical expenses are often skewed, they were converted to logarithmic form, with 2 as the base of the logarithm, before the regression models were applied. Additionally, the covariates in the analysis of the change in the average expense before and after the implementation of the IURMIS included time, age, gender and hospital level. A nonparametric test (the Wilcoxon two-sample test) was used to compare the medical expenses before and after the implementation of the IURMIS.

We used the consumer price index of China in 2009 to adjust for inflation.

## Results

### Outpatient benefits of rural residents after the implementation of the IURMIS

The county examined in this study conducted a pilot implementation of the IURMIS. The following section compares the outpatient benefits received by all rural residents before and after the implementation of the IURMIS to identify the advantages of the scheme.

#### Average expenses, compensation expenses, and rates

Table 3 shows that the average outpatient’s expenses decreased every year. The average expenses exhibited a downward trend for all three levels of hospital, except for a slight increase in county hospitals from 2010 to 2011. The amount of outpatient expenses increased with the hospital level. The differences between county and township hospitals were approximately CNY 90 over the 3 years, but the differences between township and village hospitals were only approximately CNY 20, except in 2009, when the expenses were almost the same. Regression discontinuity also showed a downward trend after the implementation of the IURMIS (Coef. = − 0.66, 95%CI:[− 0.68,-0.63], *P* < 0.001).

**Table 3:** Average outpatient expenses (in CNY) of the rural residents of the county across hospital levels in 2009, 2010, and 2011 (*n* = 20,459 in 2009, *n* = 65,258 in 2010, *n* = 59,036 in 2011)

Year	Total expenses	Expenses by hospital level		
		County	Township	Village
2009	77	152	64	62
2010	60^a^	144	60	36
2011	53^a^	148	57	32

^a^We adjust the average expenses for 2010 and 2011 based on the consumer price index in 2009 (below)

The average compensation expenses are presented in Table 4. The overall average was approximately CNY 25 in 2009, 2010, and 2011, and the values ranged from CNY 22 to CNY 26. County and township hospitals had clear increases across that period, but village clinics’ expenses decreased slightly from 2009 to 2010 and were unchanged in 2011. The highest expenses were CNY 66 in county hospitals in 2011. The township and village averages per patient were much lower and were similar to each other in 2009. However, the township compensation expenses per patient increased in 2010 and 2011, whereas the village expenses remained flat. Regression discontinuity also showed an upward trend in the compensation expenses after the implementation of the IURMIS (Coef. = 11.59, 95% CI: [11.15, 12.03], *P* < 0.001).

**Table 4:** Average compensation expenses (in CNY) of rural outpatients at county, township, and village hospitals in 2009, 2010, and 2011 (*n* = 20,459 in 2009, *n* = 65,258 in 2010, *n* = 59,036 in 2011)

Year	Per-patient compensation expenses	Per-patient compensation expenses by hospital level		
		County	Township	Village
2009	22	41	19	21
2010	26^*^	60	25	18
2011	26^*^	66	28	18

*We adjust the average expenses for 2010 and 2011 based on the consumer price index in 2009

#### Distribution of the compensation fund and patient flow

Table 5 indicates that the NRCMS compensation funds were mostly distributed to township hospitals, which accounted for 71.96% of all the funds in 2009, and received the most across all the hospital levels and years. When the IURMIS was implemented in early 2010, the compensation ratio rapidly increased from 0.22% in 2009 to 25.34% in 2011 at village clinics. However, there was little observable change at the county level; despite an increase from 27.82 to 32.18% in 2010, the compensation funds decreased to less than the 2009 percentage (27.22%) in 2011. The data showed that the outpatient medical expenses decreased significantly after the implementation of the IURMIS (Z = 57.224, *P* < 0.001).

**Table 5:** Distribution of compensation funds for rural residents in the county by hospital level in 2009, 2010, and 2011

Year	Percentage (%)			Total (%)
	County	Township	Village	
2009	27.82	71.96	0.22	100.00
2010	32.18	43.66	24.16	100.00
2011	27.22	47.44	25.34	100.00

Table 6 shows the changes in patient flow among the three hospital levels by the number of patients. In 2009, 84.99% of the compensated patients were treated at township hospitals, 14.77% were treated at county hospitals, and only 0.24% were treated at village clinics. That changed markedly in 2010, when the percentage treated at township hospitals decreased by almost half (to 47.35%) and the percentage treated at village clinics increased dramatically to 37.77%. The percentage treated at county institutions changed relatively little between 2009 and 2010. In 2011, the township and village level hospitals experienced slight increases, to 48.04% and 39.72%, respectively, and the county share decreased from 14.88 to 12.24%. This pattern suggests that, on the one hand, the healthcare demands of rural patients were met in response to the implementation of the IURMIS. On the other hand, it implies that patients tended to choose lower-level medical institutions when it seemed reasonable to them. We found the same trend in the regression discontinuity analysis (Coef. = − 0.45, 95% CI: [− 0.46, − 0.45], *P* < 0.001).

**Table 6:** Change in the outpatient flow of rural residents in the county among hospital levels between 2009 and 2011 (*n* = 20,459 in 2009, *n* = 65,258 in 2010, *n* = 59,036 in 2011)

Hospital level	Patient flow (%)		
	2009	2010	2011
County	14.77	14.88	12.24
Township	84.99	47.35	48.04
Village	0.24	37.77	39.72
Total	100.00	100.00	100.00

#### Outpatient benefits

In 2009, 56.93% of the rural patients were reimbursed for medical expenses. The percentage increased to 93.73% in 2010 but decreased to 89.25% in 2011 (Table 7). In 2009, 28.58% of the rural patients’ total medical expenses were reimbursed (Table 7). After the implementation of the IURMIS at the beginning of 2010, the percentage increased significantly, by 15.32% in 2010 and 4.97% in 2011. The higher growth rate from 2009 to 2010 demonstrated that medical demand released.

**Table 7:** Outpatient benefits of rural residents in the county in 2009, 2010, and 2011 (*n* = 20,459 in 2009, *n* = 65,258 in 2010, *n* = 59,036 in 2011)

Year	Compensation rate (%)	Reimbursement rate (%)
2009	56.93	28.58
2010	93.73	43.90
2011	89.25	48.87

The data in Tables 5, 6 and 7 lead to the conclusion that rural outpatients reasonably flow to township hospitals and village clinics, which made it easier to see a doctor (neither set of doctors was far from the patients’ residences) and to obtain higher compensation for medical expenses after the implementation of the IURMIS. Therefore, the enthusiasm of the individual users improved. This assertion can be supported as follows. First, the coverage was steady (exceeding 99%) with high fund-raising standards and subsidy projects. Second, the average expenses decreased every year. Third, the compensation rate increased every year, which was 93.73% in 2010. Fourth, the patient flow was redirected to village clinics as a reasonable response to the implementation of the IURMIS, and treatment at village clinics could decrease the medical burden and protect the health of rural residents. Moreover, the medical services and resources of the primary medical institutions may be fully utilized. This also supports the fact that hospitals of different levels have different functions.

### Analysis of the inpatient expenses of rural residents

The average expenses for the inpatient treatment of diseases (CNY 1734) were 22.75% of the total per capita net income of the rural residents in the county in 2011 (CNY 7621). Therefore, it is important to examine the average inpatient expenses, the number of hospitalization days, and the distribution of expenses to understand the functions of the IURMIS for reducing the burden of medical expenses in rural areas.

#### Average expenses and the number of hospitalization days

In China, all of the patients can access any kinds of institutions, but different hospital levels have different functions. That is to say, preventive care, minimal health care and rehabilitation services are always treated in primary hospitals such as township hospitals and county hospitals, but severe acute and complicated cases are always treated in tertiary hospitals such as city and provincial hospitals. Therefore, we divided the hospitals into four categories in this section.

Table 8 sets forth the average medical expenses overall and by hospital level for inpatient services. Hospitalizations in county hospitals ranked first (64.80%), followed by township hospitals (33.03%) and city and provincial hospitals (2.17%). The numbers in city and provincial hospitals are so low, it is because that the patients in our study were rural residents and most were common diseases, and they prefer to get treatment in lower medical institutions. Beyond that, lower institutions have higher reimbursement rates based on the above analysis.

**Table 8:** Numbers and percentages of cases, the average inpatient expenses, and the length of hospitalization of rural residents at four levels of medical institutions in 2011 (*n* = 3500)

Hospital level	Number of cases (%)	Average expenses		Number of hospitalization days	
		Mean (SD)	Median	Mean (SD)	Median
Township	1156 (33.03)	894 (1576)	730	8.6 (2.6)	8
County	2268 (64.80)	2165 (2128)	1673	10.5 (6.0)	9
City	21 (0.60)	5113 (4359)	3500	10.1 (7.1)	8
Provincial	55 (1.57)	21,493^*^ (42087)	9075	16.8 (19.4)	13
Total	3500	2067 (6136)	1188	10.0 (5.8)	8

^a^The average expenses in provincial hospitals were much greater because there was a patient with duodenal neoplasms, who received surgical treatment and was in hospital for 131 days. His hospital expenses were CNY 300, 931, which was the highest value of the provincial inpatients. The lowest value was CNY 875 in the same year; therefore, the SD of the provincial expenses was also large

Furthermore, we calculated the average expenses (CNY 2067) and the number of hospitalization days (10.0) for all hospitals, both of which were less than the values obtained in the Chinese Fourth National Health Services Survey (CNY 3412 and 10.3, respectively) [23].

## Discussion

In a word, the implementation of the IURMIS in the county resulted in a decrease in the medical burden in rural areas. These effects are as follows:

1. Complete medical insurance coverage of urban and rural residents has been realized, and there is no residence-based coverage gap. First, the integration of the URBMI and the NRCMS conformed to the “majority rule”, which increased the number of insured people and the insurance funds. Integrating medical insurance can enhance its stability because the population tends to be younger in rural areas than in urban areas, and younger populations tend to be healthier [26, 27]. This is helpful for increasing the medical insurance funds for current and future conditions (The Sixth National Census).
2. The implementation of the IURMIS decreased the medical financial burden and increased rural residents’ benefits. In 2010, the average inpatient expenses (which were controlled within CNY 1991 at the designated county level hospitals) were approximately CNY 735 at township hospitals, which was lower than the local average standard. Shaanxi Province had the top average inpatient reimbursement rate 67.5% [28]. In 2011, the average inpatient expenses were lower at designated township and county hospitals than in other areas of Shaanxi Province in this study; they were CNY 2165 and CNY 894, respectively.
3. The primary medical resources (county-level and township-level hospitals) were well utilized because of the implementation of the IURMIS. In 2010, the number of people served in primary medical institutions was approximately 0.65 million, and the reimbursement rate was 43.90%. The benefit rate for outpatients rapidly increased from 28.58% in 2009 to 48.87% in 2011 in this study. These findings are in accordance with the results of the study by the People’s Government of Feng County in 2011.

With the above analysis, firstly, primary medical institutions are tasked with providing preventive care, minimal healthcare and rehabilitation services. Secondly, the higher institutions have lower reimbursement rates, but primary institutions have higher reimbursement rates. This is intended to distribute people with minor illnesses to primary institutions. Therefore, rural patients, who were the main group in our study, flowed from higher-level to lower-level medical institutions after the implementation of the IURMIS, increasing the level of compensation funds at the lower-level medical institutions. In other words, rural patients used village clinics (which are the medical institutions for village units) and township hospitals reasonably. The different types of outpatient care at the two levels of facilities also indicate the different functions of the hospital levels.

Thirdly, the primary institutions were near the residences of the rural patients, which made doctor visits more convenient. Moreover, they could save more time and money, which partially decreased their medical expenses. Hence, more and more rural residents choose the lower-level medical institutions, which lead to an increase in utilization and a resultant decrease in medical costs. These findings are not only consistent with the data for 2012 from the Chinese Health Statistics Yearbook [24], which suggested that rural residents reasonably flow between village clinics and higher-level medical institutions, but also indicate that health resources in primary medical institutions were more completely utilized.

## Conclusions

Between the implementation of the IURMIS in Feng County at the beginning of 2010 and November, 2011, there were 0.805 million participants, coverage of the NRCMS stabilized at more than 98% (up to 95% among urban residents), and complete medical insurance coverage for urban and rural residents was realized [28]. However, implementation of the IURMIS will inevitably face the following challenges:

1. Absence of institutional design and guidelines from the national government. At present, the social security department administrates the URBMI, but the health departments manage the NRCMS in most areas of China. The division of management will increase the difficulties for the IURMIS.
2. Inadequate administrative capacity to operate the schemes. In the process of the IURMIS, there are many problems such as a lack of staff, insufficient funds and low quality staff. The shortage of staff with the requisite professional qualifications and special training will seriously affect the efficiency and quality of the IURMIS.
3. The requirement of a management system that responds to such integration in a timely and efficient manner. Because the medical insurance information is still being developed, and due to the division of various information network systems, it is difficult for the various information sources to communicate with each other.
4. Policymakers should find a balance between the healthcare providers and participants. Fear of impaired health benefits and the differences between the URBMI and NRCMS in aspects such as payment standards, benefit packages, etc., were found to be the main reason for opposing the IURMIS [29]. Therefore, the key to the coordination of urban and rural healthcare insurance lies in equal financing and equal benefits.

Combining the suggestions above, despite the challenges ahead, a new integrated healthcare scheme is promising and could be applied in the near future. What can other regions in China learn? On the one hand, the government could expand the reform of public hospitals at the county level and strengthen its investments and infrastructure in rural medical institutions at the local (village) level. On the other hand, it is important for policymakers to find balance between the providers and participants, especially for weak areas of economic development.

Medical care insurance is an important component of the social guarantee system. It is also a complicated item among the various insurance systems; therefore, it is called a “international problem”. This analysis, firstly, increases the understanding of the different Chinese medical insurance schemes and their development. Secondly, the implementation of the IURMIS is not only the inevitable result of China’s basic medical development but also part of China’s urbanization process. The implementation of the IURMIS meant that rural residents had the same opportunities to receive medical services that were equal to those of urban residents. Thus, the IURMIS promotes the integration of urban and rural areas, and can serve as a model for other developing countries [30–32].

Despite its strengths, this study had several limitations. First, although the data covered 2009 through 2011, to assess changes to the average expenses of rural residents before and after the implementation of the IURMIS, our argument is not strong because of the short period covered by the data, particularly the inpatient data, which were only collected for 1 year (2011). Further studies that cover longer periods could strengthen these assertions. Unfortunately, this study has taken this particular analysis as far as possible because of database corruption and a lack of comparable datasets. Second, our results cannot be generalized to other districts in China because of regional differences. However, after the implementation of the IURMIS in China, the reimbursement rate has improved and medical expenses have decreased in many other pilot districts. As an underdeveloped district in Shaanxi, Feng County can make the best decision in light of its own level of economic and social development to best protect public health.

## Abbreviations

IURMIS: Integration of Urban and Rural Medical Insurance Scheme
NRCMS: New Rural Cooperative Medical System
RD: Regression Discontinuity
TH: Township Hospital
URBMI: Urban Residents Basic Medical Insurance
